# Screening Model for Bladder Cancer Early Detection With Serum miRNAs Based on Machine Learning: A Mixed‐Cohort Study Based on 16,189 Participants

**DOI:** 10.1002/cam4.70338

**Published:** 2024-10-23

**Authors:** Cong Lai, Zhensheng Hu, Jintao Hu, Zhuohang Li, Lin Li, Mimi Liu, Zhikai Wu, Yi Zhou, Cheng Liu, Kewei Xu

**Affiliations:** ^1^ Department of Urology, Sun Yat‐sen Memorial Hospital Sun Yat‐sen University Guangzhou Guangdong China; ^2^ Guangdong Provincial Key Laboratory of Malignant Tumor Epigenetics and Gene Regulation, Sun Yat‐sen Memorial Hospital Sun Yat‐sen University Guangzhou Guangdong China; ^3^ Department of Medical Informatics, Zhongshan School of Medicine Sun Yat‐sen University Guangzhou Guangdong China; ^4^ Guangdong Provincial Clinical Research Center for Urological Diseases Guangzhou Guangdong China; ^5^ Sun Yat‐sen University School of Medicine Sun Yat‐sen University Shenzhen Guangdong China

**Keywords:** bladder cancer, early detection, machine learning, screening model, serum miRNA

## Abstract

**Background:**

Early detection of bladder cancer (BCa) can have a positive impact on patients' prognosis. However, there is currently no widely accepted method for early screening of BCa. We aimed to develop an efficient, clinically applicable, and noninvasive method for the early screening of BCa by detecting specific serum miRNA levels.

**Methods:**

A mixed‐cohort (including BCa, 12 different other cancers, benign disease patients, and health population) study was conducted using a sample size of 16,189. Five machine learning algorithms were utilized to develop screening models for BCa using the training dataset. The performance of the model was evaluated using receiver operating characteristic curve and decision curve analysis on the testing dataset, and subsequently, the model with the best predictive power was selected. Furthermore, the selected model's screening performance was evaluated using both the validation set and external set.

**Results:**

The BCaS3miR model, utilizing only three serum miRNAs (miR‐6087, miR‐1343‐3p, and miR‐5100) and based on the KNN algorithm, is the superior screening model chosen for BCa. BCaS3miR consistently performed well in both the testing, validation, and external sets, exceeding 90% sensitivity and specificity levels. The area under the curve was 0.990 (95% CI: 0.984–0.991), 0.964 (95% CI: 0.936–0.984), and 0.917 (95% CI: 0.836–0.953) in the testing, validation, and external set. The subgroup analysis revealed that the BCaS3miR model demonstrated outstanding screening accuracy in various clinical subgroups of BCa. In addition, we developed a BCa screening scoring model (BCaSS) based on the levels of miR‐1343‐3p/miR‐6087 and miR‐5100/miR‐6087. The screening effect of BCaSS is investigated and the findings indicate that it has predictability and distinct advantages.

**Conclusions:**

Using a mixed cohort with the largest known sample size to date, we have developed effective screening models for BCa, namely BCaS3miR and BCaSS. The models demonstrated remarkable screening accuracy, indicating potential for the early detection of BCa.

## 1 Introduction

Bladder cancer (BCa) has an annual incidence of over 0.57 million cases and results in at least 0.21 million deaths according to global cancer statistics [1]. BCa can be categorized based on whether the tumor invades the muscle layer of the bladder into nonmuscle invasive BCa (NMIBC), which encompasses Ta, Tis, and T1, and muscle‐invasive BCa (MIBC) (≥ T2). The 5‐year survival rate of NMIBC is as high as 95.4% for Ta and Tis, and 88% for T1. In contrast, MIBC has a 5‐year survival rate that varies from 69.4% for T2 to 4.8% for T4 [2, 3]. Therefore, early screening and detection of BCa are crucial for improving treatment outcomes and enhancing patient prognosis.

Cystoscopy and urine cytology are considered the gold standards for BCa detection. However, while cystoscopy is an invasive procedure that necessitates specialized sterilized instruments and can result in complications such as pain and hematuria, it has low sensitivity for identifying flat BCa and depends on the physician's experience [4]. Urine cytology validation can yield false negative outcomes due to the inadequate of cast‐off cells in urine. Obtaining a more reliable diagnosis necessitates a substantial volume of urine samples collected numerous times. Furthermore, outcomes from urine cytology can be influenced by factors such as urinary tract infections and hematuria [5, 6]. Therefore, neither cystoscopy nor urine cytology validation is suitable for population‐based BCa screening. A practical, cost‐effective, noninvasive, and reliable early screening method is urgently required to accomplish early BCa detection and improve patient survival prognosis.

Liquid biopsy has become an important tool for early screening, detection and disease monitoring of tumors due to its noninvasive nature. MicroRNAs (miRNAs) are present in bodily fluids, including serum, tissue fluid, and urine, and have proven their effectiveness in tumor screening and detection [7, 8]. Nevertheless, there are many unavoidable issues in using urine miRNA for diagnosing BCa. The amount of miRNA in urine is affected by the number of cast‐off epithelial cells in the urinary tract, the volume of the urine sample, and the influence of urinary tract infections and hematuria, making quality control of urine miRNA detection samples difficult to standardize and limiting the clinical application of urine miRNA in diagnosing BCa. In contrast, serum miRNA provides advantages that cannot be compared with urine. The stability of miRNA levels in serum is high, and it is unaffected by urinary tract symptoms related to BCa. Furthermore, obtaining, storing, and transporting serum samples for miRNA detection is a convenient process, and there are already established protocols for serum miRNA detection [6, 9]. Consequently, serum miRNA is practical for large‐scale population screening of BCa.

Although there have been only a few studies on serum miRNA‐based BCa detection, some have demonstrated high sensitivity and specificity. For instance, one such study developed a model using six serum miRNAs based on 250 BCa patients and 240 controls, achieving an area under (AUC) the receiver operating characteristic curve (ROC) of 0.899 [9]. Likewise, another study identified seven miRNAs as BCa diagnostic markers from 972 cases and constructed a detection model with 95% sensitivity using Fisher's linear discriminant analysis [10]. In addition, Yu et al. [11] reported an AUC of 0.899 for a detection model consisting of four serum miRNAs based on 112 BCa patients and 112 controls. However, the effectiveness of using serum miRNA models has not yet been established due to factors such as inadequate sample sizes. Besides, the lack of diversity among study samples, which only include cases and controls of BCa but no other diseases or types of cancers, may impede the effectiveness of using these models in early‐stage tumor screening.

Our study aimed to develop an efficient screening model for BCa that utilizes serum miRNAs derived from a mixed cohort of 16,189 samples. Our study stands out from previous works due to our large sample size, which comprises the most diverse array of cancer and benign disease types. We employed five different machine learning algorithms to select potential candidate serum miRNAs and to construct a screening model that can distinguish BCa in a mixed cohort. Additionally, we evaluated the screening efficiency of the model through testing set and validation set and subgroup analysis, demonstrating its potential for early‐stage BCa screening.

## 2 Materials and Methods

### 2.1 Data Collection and Processing

In this study, we acquired one serum miRNA sequencing datasets (GSE211692) from the Gene Expression Omnibus Database (https://www.ncbi.nlm.nih.gov/geo/). Perl software (https://www.perl.org/) was used to convert the miRNA data matrix IDs for subsequent data analysis. According to the ratio of 4:1, the dataset was randomly divided into model construction set and validation set. Model construction set was randomly divided into training set and testing set. However, the ratio of nonbladder cancer (NBCa) samples to BCa samples in this dataset was highly imbalanced (39.6:1), which could significantly impact the performance of the classification model, particularly by biasing it toward the majority class. To mitigate this, we implemented data balancing using the BorderlineSMOTE method, an improved oversampling algorithm based on synthetic minority oversampling technique (SMOTE) [12]. BorderlineSMOTE improves upon standard SMOTE by focusing on the Danger samples‐minority class samples that are near the decision boundary between classes and more likely to be misclassified. This method divides the minority class into three categories: Safe, Danger, and Noise. Only the Danger samples are oversampled, which avoids generating redundant samples from easily classified regions (Safe) or adding noise by oversampling from noisy areas (Noise). This targeted approach ensures that the minority class distribution is improved in areas where classification is most challenging, which enhances the model's ability to distinguish between BCa and NBCa. After applying BorderlineSMOTE, we ensured that the balanced dataset was used in the training phase to improve the model's generalization to the minority class while preserving the distribution characteristics of the majority class.

Furthermore, the entire dataset was divided into training and test sets using stratified sampling based on disease categories. This ensured that the distribution of BCa, NBCa, and OCa samples in the test set remained consistent with the original dataset distribution. By maintaining the original distribution of disease categories in the test set, we ensured that the model's performance evaluation reflects its generalization ability across real‐world data distributions. The stratified sampling approach also allowed us to account for the class imbalance while still preserving the integrity of the dataset.

### 2.2 Identification of Candidate miRNA Features for BCa Screening Model

To explore potential specific biomarkers for early screening of BCa, it is necessary to consider that not all miRNAs are associated with detection indicators of BCa and that some miRNAs display redundancy in their expression levels. Therefore, to identify miRNA features with maximum information value and relevance to BCa screening, it is crucial to minimize feature redundancy and eliminate irrelevant biomarkers. In this study, we employed various methods, including Maximum Relevance Minimum Redundancy algorithm (MRMR), Information Value algorithm (IV), Mutual Information method, and Tree model screening, to conduct candidate feature identification on the training dataset. These methods focused on selecting feature subsets based on the expression level differences of miRNA features in BCa and NBCa samples.

### 2.3 BCa Screening Model Was Constructed Based on Machine Learning Algorithms

Given the limited screening efficacy of a single miRNA feature for BCa in complex data situations, combining multiple miRNA features through linear or nonlinear relationships can achieve efficient and accurate diagnosis of BCa. Therefore, we utilized five different machine learning algorithms, including K‐Nearest Neighbor (KNN), Support Vector Machine (SVM), eXtreme Gradient Boosting (XGBT), Random Forest (RF), and Logistic Regression (LR), and validated the identified miRNA feature subsets using five‐fold crossvalidation methods to find the best combination and build models. Specifically, the five machine learning algorithms were used to create different BCa detection classifiers consisting of 1–5 miRNAs based on training set data. We evaluated the BCa screening prediction model's AUC, sensitivity (SEN), specificity (SPE), accuracy (ACC), and negative predictive value (NPV) based on the ROC curve of testing set to obtain the best miRNA combination. We apply five machine learning methods and use five‐fold crossvalidation method to model based on the best combination, selected the best model based on the criteria that the AUC, SEN, SPE, ACC, and NPV were high, and the model's miRNA feature subset was relatively small.

### 2.4 The Evaluation of the Effectiveness of a BCa Screening Model

We using validation cohorts to calculate the ROC curve of the BCa screening model for the AUC, SEN, SPE, ACC, and NPV. These metrics are particularly important for addressing the class imbalance in the dataset, with AUC, SEN, and SPE helping to evaluate the model's ability to correctly identify both BCa and non‐BCa cases, reducing bias toward the majority class. We also compared the net benefit of the model and each serum miRNA by utilizing decision curve analysis (DCA) to assess the clinical utility of the model by weighing the benefits of true positives against the costs of false positives in an imbalanced context. In addition, we evaluated the model's discrimination index across different disease groups, including BCa, 12 other cancer patients (OCa), benign disease patients (BDs), and healthy participants (HPs) in both the test and validation sets. This analysis aimed to determine whether the model effectively distinguishes BCa patients from other disease groups. Furthermore, we examined the model's detection accuracy across various clinical subgroups of BCa patients to assess its reliability in different clinical contexts. Finally, external dataset validation is conducted to further substantiate the aforementioned results.

### 2.5 Establishment and Evaluation of miRNA Scoring Model for BCa Screening

However, since miRNAs expression level are not normalized, sequencing analysis variations and technical errors could affect the results and lead to variability in the parameters or coefficients associated with each miRNA. To address this issue and provide an adequate normalization factor for serum samples, we proposed utilizing a combination (as a ratio) of an overexpressed and an underexpressed miRNA within the same RNA sample from a BCa patient as a suitable within‐sample normalization technique. In this study, the BCa screening score model (BCaSS model) was established based on the miRNAs features of the screening model established in above. Specifically, the scores for BCa patients were calculated based on the ratio of high‐expression miRNAs to low‐expression miRNAs, and five machine learning algorithms were utilized for constructing the BCa screening models. The performance of each model was evaluated by calculating the AUC, ACC, SEN, SPE, and NPV values, and the machine learning model with the best screening efficacy was selected as the BCaSS model. Testing set and validation set were used to evaluate the screening effectiveness of the BCaSS model. Finally, external dataset validation is conducted to further substantiate the aforementioned results.

### 2.6 Statistical Analysis

We utilized Python version 3.10 (https://www.python.org/) and Scikit‐Learn 1.2 (https://scikit‐learn.org/stable/) for machine learning algorithm modeling and principal component analysis (PCA). To assess the screening efficiency of the model, we drew ROC curve and calculated AUC value, as well as ACC, SEN, SPE, and NPV. The nonpaired *t*‐test was employed for comparing continuous variables between two groups, and *p* < 0.05 was regarded as statistically significant.

## 3 Results

### 3.1 Participants and Study Design

**FIGURE 1:**
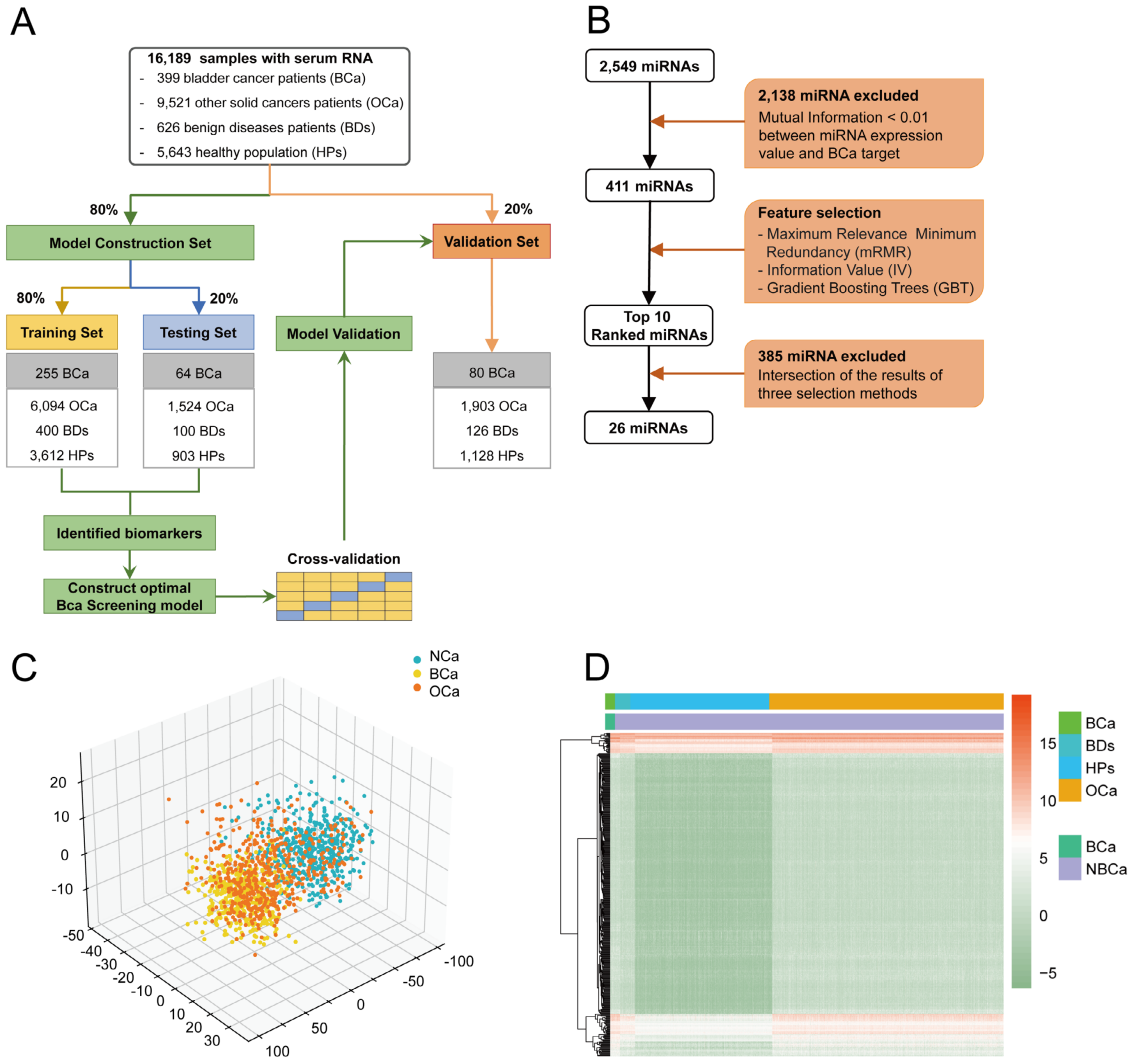
Select candidate miRNAs for BCa screening. (A) Work flow for establishing the screening model using 16,189 samples. (B) Flowchart for selecting candidate miRNA. (C) Principal component analysis map using 411 miRNAs features. (D) Hierarchical clustering analysis of a heatmap showing 411 miRNAs features. The color scale represents the miRNA expression level.

To ensure the specificity, robustness, and universality of identifying BCa for screening, we enrolled patients with BCa, 12 OCa, BDs, and HPs, forming a large mixed cohort of 16,189 samples (Table 1). The samples with undefined disease types were excluded during data preprocessing. According to the ratio of 4:1, the dataset was randomly divided into model construction set (*n* = 12,952) and external validation set (*n* = 3237). Model construction set was randomly divided into training set (*n* = 10,361) and testing set (*n* = 2591) through five‐fold crossvalidation (Figure 1A).

**TABLE 1:** Information of 16,189 participants mixed cohort.

Profiling data		Training set, *n* = 10,361	Testing set, *n* = 2591	Validation set, *n* = 3237
Bladder cancer, *n* Median age, years		255; 67.61 (32–93)	64; 66.37 (31–90)	80; 68.58 (39–89)
Other cancers		6094	1524	1903
1	Lung cancer, *n* Median age, years	1087 65.53 (26–88)	272 64.92 (30–86)	340 65.05 (35–86)
2	Colorectal cancer, *n* Median age, years	1021 63.83 (20–93)	256 63.66 (22–90)	319 64.49 (35–94)
3	Gastric cancer, *n* Median age, years	907 65.03 (20–90)	227 65.49 (21–88)	284 65.49 (20–90)
4	Prostate cancer, *n* Median age, years	658 67.31 (39–92)	164 67.53 (44–87)	205 68.23 (51–88)
5	Pancreatic cancer, *n* Median age, years	545 64.54 (32–86)	136 63.78 (27–84)	170 65.40 (23–89)
6	Breast cancer, *n* Median age, years	432 55.61 (29–86)	108 54.20 (27–87)	134 54.11 (30–79)
7	Esophageal cancer, *n* Median age, years	362 66.56 (40–90)	91 66.23 (49–87)	113 66.60 (37–90)
8	Biliary tract cancer, *n* Median age, years	258 65.91 (30–89)	64 65.92 (43–86)	80 66.87 (26–85)
9	Ovarian carcinoma, *n* Median age, years	256 56.52 (16–82)	64 56.10 (31–82)	80 55.73 (27–79)
10	Hepatocellular carcinoma, *n* Median age, years	223 67.78 (41–85)	55 66.90 (45–86)	70 67.17 (31–89)
11	Sarcoma, *n* Median age, years	191 47.14 (1–97)	48 45.75 (8–89)	60 46.93 (11–88)
12	Glioma, *n* Median age, years	154 56.50 (17–87)	39 53.76 (14–82)	48 53.91 (17–84)
Benign diseases, *n* Median age, years		400 53.66 (3–85)	100 52.41 (12–85)	126 55.13 (6–81)
Healthy population, *n* Median age, years		3612 67.18 (20–98)	903 67.75 (31–93)	1128 67.29 (35–100)

### 3.2 Selection of Serum miRNAs as Candidate Markers for BCa Screening

In the selection of serum miRNAs as candidate markers for BCa screening, we employed a multistage feature selection process to ensure robustness and interpretability. First, we used the mutual information algorithm to evaluate the correlation between miRNA expression levels and BCa screening results based on the training set data. Mutual information measures the dependency between variables, and miRNAs with a mutual information score below 0.01 were considered to have negligible relevance to BCa and were excluded from further analysis. This threshold was chosen to remove features with minimal predictive power. Consequently, 2138 miRNAs were filtered out from the initial feature pool (Figure 1B). Following this step, the remaining 411 miRNA features underwent dimensionality reduction using principal component analysis (PCA) to map the high‐dimensional data into a lower‐dimensional space while retaining as much variance as possible. This process was critical for reducing redundancy and enhancing the model's efficiency. We visualized the differential expression of these miRNAs using a heatmap in conjunction with unsupervised clustering analysis (Figure 1C,D), which revealed that while these 411 features effectively differentiated between BCa and NBCa groups, they did not successfully distinguish between BCa and OCa groups. To further refine the miRNA features specific to BCa, we implemented a combination of three feature selection algorithms: MRMR, IV and a tree‐based embedding algorithm. MRMR was employed to prioritize features that had high relevance to BCa while minimizing redundancy with other selected features. IV was utilized to assess the predictive power of each feature, and the tree‐based embedding method integrated feature importance scores from decision‐tree models. This multialgorithm approach ensured that we retained features with both high predictive accuracy and interpretability. The top 10 miRNA features from each algorithm were selected in an iterative process, resulting in a final set of 26 candidate miRNA features. The expression levels of these 26 miRNAs were statistically significantly different between BCa and NBCa groups, as demonstrated by subsequent analysis (Figure S1). This final set of miRNAs provides a strong basis for developing a robust BCa screening model.

### 3.3 Determination of the Optimal miRNA Combination and Machine Learning Model for Early Screening of BCa

**FIGURE 2:**
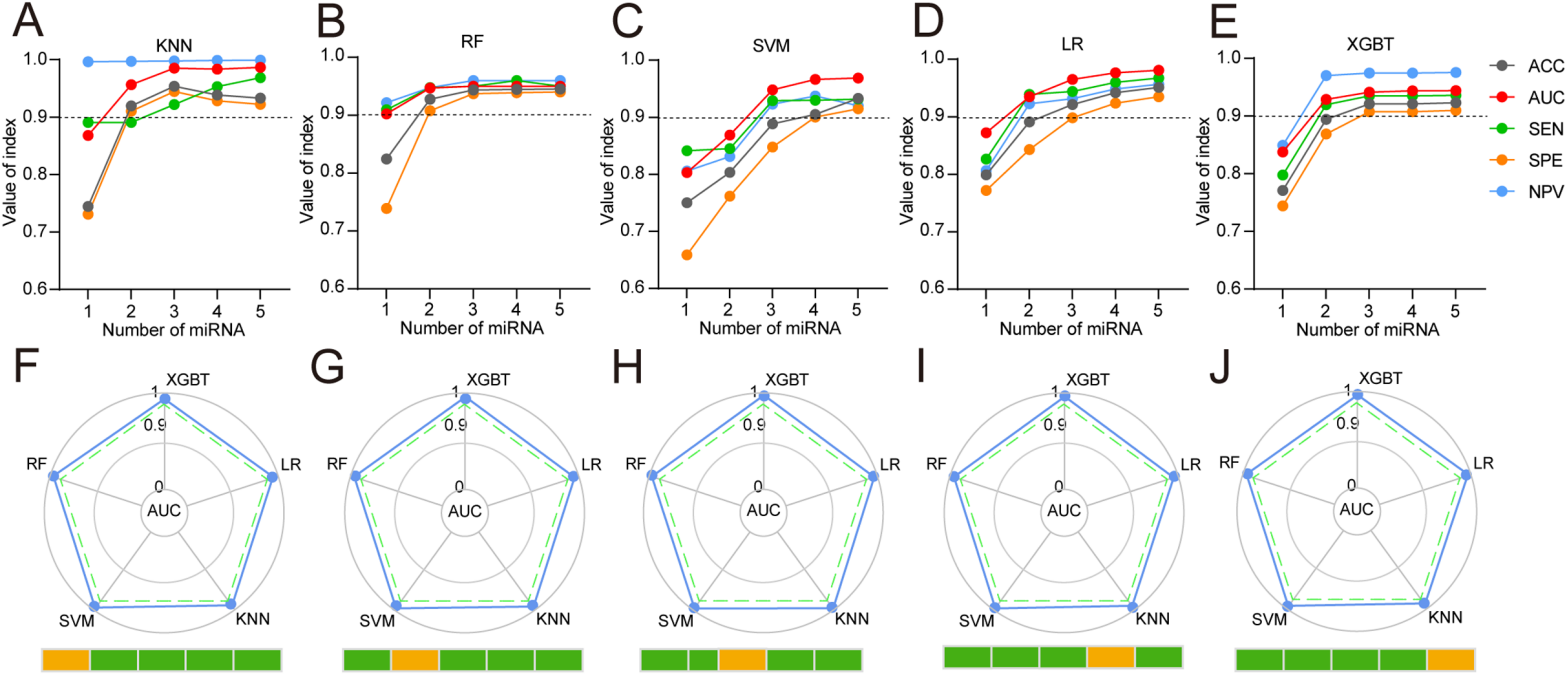
Establish the best screening model of BCa. The ACC, SEN, SPE, and NPV of the BCa screening models established using 1–5 miRNAs based on the KNN (A), RF (B), SVM (C), LR (D), and XGBT (E) algorithms, respectively, in the testing set. (F–J) The radar chart summarized the five‐fold crossvalidation results of the AUC for three miRNA model to recognize BCa in the testing set.

**FIGURE 3:**
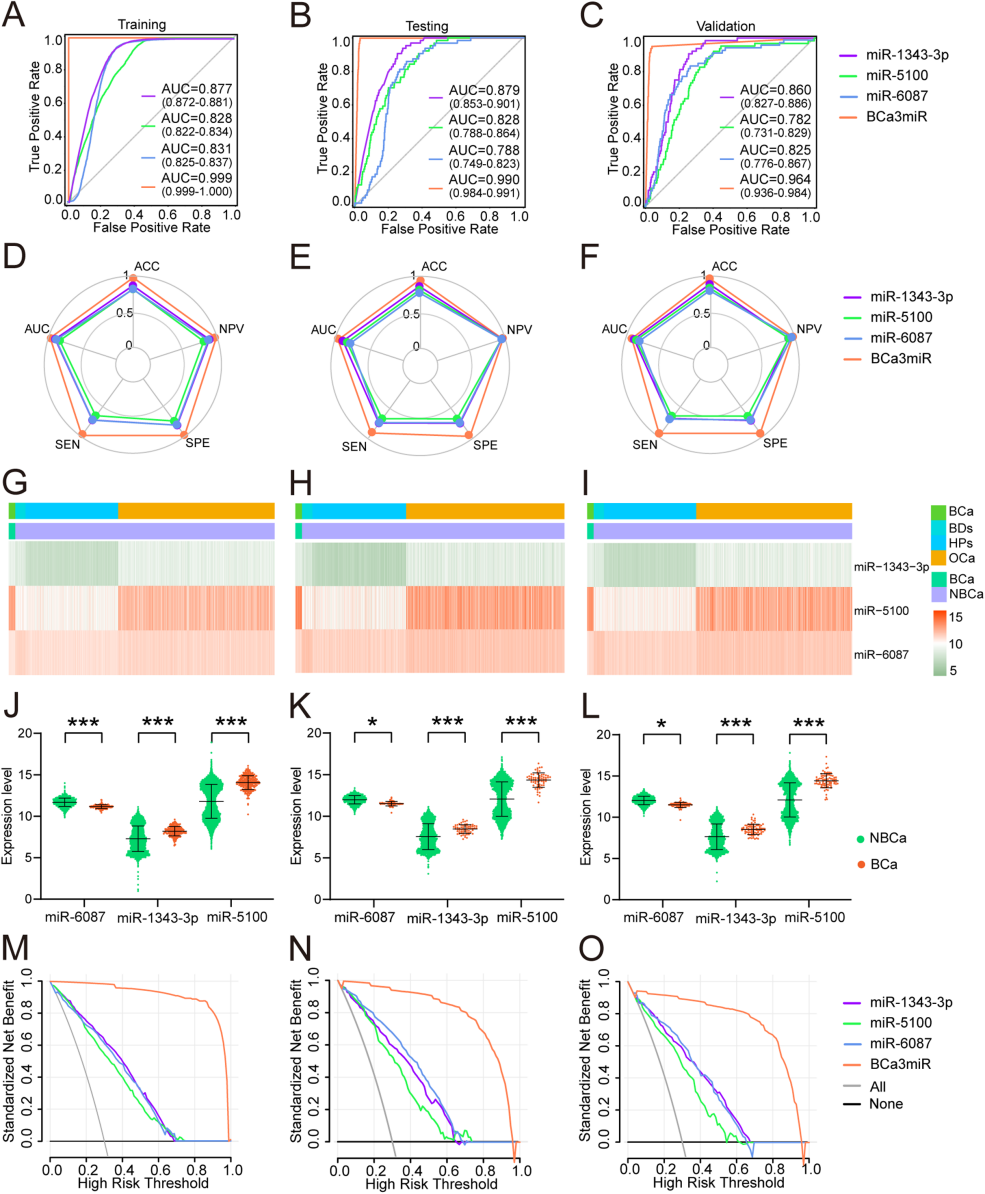
Effectiveness of the BCa screening model. ROC curves of BCaS3miR model in training set (A), testing (B), and validation set (C). The radar chart summarized the ability of BCaS3miR model to recognize BCa in training set (D), testing set (E), and validation set (F), which were determined by AUC, SEN, SPE, ACC and NPV. Heatmaps of three miRNAs in training set (G), testing set (H), and validation set (I). The levels of miR‐1343‐3p, miR‐5100, and miR‐6087 in the BCa group and NBCa group in training set (J), testing set (K) and validation set (L). In a wide range of decision threshold probability, the difference of net benefit between BCaS3miR model and serum biomarkers using the DCA in the validation set (M), testing set (N), and validation set (O).

Based on 26 candidate miRNA features, five different machine learning algorithms (including KNN, SVM, XGBT, RF and LR) were used to design classifiers containing 1–5 miRNAs to identify the optimal miRNA combination and establish a screening model. The broken line graph shows the mean ACC, AUC, SEN, SPE, and NPV of screening models established based on 1–5 miRNAs using five machine learning algorithms (Figure 2A–E). It can be seen that the BCa screening model constructed using three miRNA features meets the requirements of excellent predictive efficiency and a small number of features. Finally, it was found that the screening model composed of three miRNAs, miR‐1343‐3p, miR‐5100, and miR‐6087, had the best predictive efficacy. The radar chart of the results of five‐fold crossvalidation shows that using three miRNA features to establish a BCa screening model based on five machine learning algorithms can achieve stable and excellent screening performance (Figure 2F–J, Table S1). Comparing the ACC, AUC, SEN, SPE, and NPV of three serum miRNAs feature of five machine learning algorithms model, the best BCa screening model was established based on the KNN algorithm (BCaS3miR model), and the screening index was KNeighbors Classifier (*n*_neighbors = 120, algorithm = ‘auto’, weights = ‘distance’, *p* = 2, leaf_size = 30, *n*_jobs = 60). The AUC of the training set, testing set and validation set (Figure 3A–C) were 0.999 (95% CI: 0.999–1.000), 0.990 (95% CI: 0.984–0.991) and 0.964 (95% CI: 0.936–0.984), respectively. The radar chart shows the AUC, SEN, SPE, ACC, and NPV of the BCaS3miR model in the training set, testing set, and validation set (Figure 3D–F, Table S2). The heatmap clustering shows the expression levels of these three miRNAs in each sample (Figure 3G–I). A scatter plot comparing the expression levels of these three miRNAs between BCa and NBCa shows significant differences (Figure 3J–L). It can be seen that miR‐1343‐3p and miR‐5100 were all highly expressed in the serum of BCa patients, while miR‐6087 was low expressed in the serum of BCa patients. In the DCA, BCaS3miR model demonstrated an absolute superiority net benefit within a wide range of decision‐making threshold probabilities, compared to the miR‐6087, miR‐1343‐3p, and miR‐5100 in the training set, testing set, and validation set (Figure 3M–O). Therefore, the BCaS3miR model has excellent screening performance and exceptional prediction stability.

### 3.4 Discrimination of BCa and OCa, BDs, HPs Using the BCaS3miR Model

**FIGURE 4:**
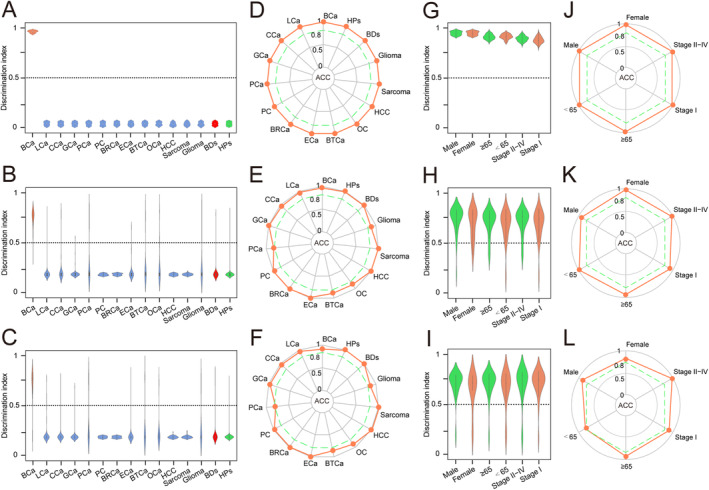
Screening performance and subgroup analysis of the BCaS3miR model. Discrimination index was calculated and plotted in a dot plot among BCa, 12 different OCa, BDs, and HPs for the discriminants in training set (A), testing set (B) and validation set (C) Discrimination index ≥ 0.5 indicated BCa and discrimination index < 0.5 indicated NBCa. The radar chart summarized the ACC of each cancer type, red polyline represented the ACC value of BCaS3miR model in distinguishing each cancer in training set (D), testing set (E), and validation set (F). Discrimination index was calculated and plotted in a dot plot among BCa in different clinical subgroups for the discriminants in training set (G), testing set (H), and validation set (I). The radar chart summarized the ACC of each subgroup, red polyline represented the ACC value of BCaS3miR model in distinguishing each subgroup in training set (J), testing set (K), and validation set (L).

To determine whether the serum BCaS3miR model can distinguish bladder cancer from OCa (LCa, lung cancer; CCa, colorectal cancer; GCa, gastric cancer; PCa, prostate cancer; PC, pancreatic cancer; BRCa, breast cancer; ECa, esophageal cancer; BTCa biliary tract cancer; OC, ovarian cancer; HCC, hepatocellular carcinoma; Sarcoma; Glioma), BDs and HPs, we calculated the discrimination index of the BCaS3miR model in different diseases in training set (Figure 4A), testing set (Figure 4B), and validation set (Figure 4C). A discrimination index ≥ 0.5 was determined as BCa, and a discrimination index < 0.5 was determined as NBCa. The results showed that the BCaS3miR model has excellent discrimination ability for BCa and other 12 tumors, BDs, and HPs. The results of the screening ACC were presented as a radar chart (Figure 4D,F, Table S3). The BlaS3miR model exhibited a detection ACC of more than 80% in distinguishing BCa from 12 OCa, BDs, and HPs.

### 3.5 Subgroup Analysis of the BCaS3miR Screening Model

To determine the screening accuracy of the BCaS3miR model in different clinical subgroups of BCa, we calculated the discrimination index of the BCaS3miR model in male and female, age < 65 and age ≥ 65, Stage I and Stage II–IV subgroups in training set (Figure 4G), testing set (Figure 4H), and validation set (Figure 4I). The BCaS3miR model showed excellent screening performance in different clinical subgroups of BCa. The radar chart (Figure 4J–L, Table S4) presents the results of the screening ACC, indicating that the BlaS3miR model's detection ACC is greater than 80% for different clinical subgroups.

### 3.6 Establishment of BCa Screening Score Model

This study identified miRNAs including miR‐1343‐3p, miR‐5100, and miR‐6087 as potential screening markers for BCa. The first two were over expressed in the serum of BCa patients, while miR‐6087 was under expressed. The ratio of high‐expressed and low‐expressed miRNAs was used as a score, and five different machine learning algorithms were applied to establish the BCa screening score model by applying the values of miR‐1343‐3p/miR‐6087 and miR‐5100/miR‐6087. Using the ratio model effectively alleviated the batch effects caused by sequencing, making it more suitable for clinical application. Finally, the best BCa screening score model (BCaSS) was established based on the KNN algorithm.

**FIGURE 5:**
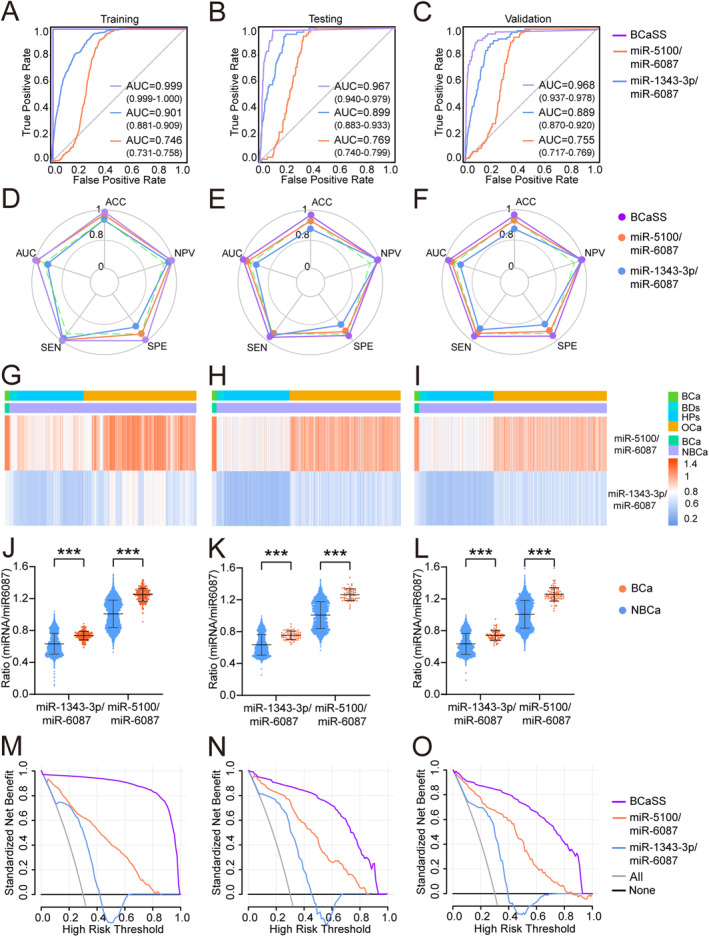
Establish the bladder cancer screening score model. ROC curves of BCaSS model in training set (A), testing (B), and validation set (C). The radar chart summarized the ability of BCaSS model to recognize BCa in training set (D), testing set (E), and validation set (F), which were determined by AUC, SEN, SPE, ACC, and NPV. Heatmaps of the values of miR‐1343‐3p/miR‐6087 and miR‐5100/miR‐6087 in training set (G), testing set (H), and validation set (I). The values of miR‐1343‐3p/miR‐6087 and miR‐5100/miR‐6087 in the BCa group and NBCa group in training set (J), testing set (K), and validation set (L). In a wide range of decision threshold probability, the difference of net benefit between BCaSS model and serum biomarkers using the DCA in the validation set (M), testing set (N), and validation set (O).

The AUC value of training set (Figure 5A), testing set (Figure 5B), and validation set (Figure 5C) were 0.999 (95% CI: 0.999–1.000), 0.967 (95% CI: 0.940–0.979), and 0.968 (95% CI: 0.937–0.978). The radar chart displayed the AUC, SEN, SPE, ACC, and NPV of BCaSS model in the training set, testing set, and validation set (Figure 5D,F, and Table S5). The heatmap clustering shows the values of miR‐1343‐3p/miR‐6087 and miR‐5100/miR‐6087 in each sample (Figure 5G–I). A scatter plot comparing the values of miR‐1343‐3p/miR‐6087 and miR‐5100/miR‐6087 between BCa and NBCa shows significant differences (Figure 5J–L). In the DCA, BCaSS model demonstrated an absolute superiority net benefit within a wide range of decision‐making threshold probabilities compared with the values of miR‐1343‐3p/miR‐6087 and miR‐5100/miR‐6087 (Figure 5M–O). These results suggest that the BCaSS model has excellent predictive efficacy and stability in both the training set and validation set.

### 3.7 Screening Performance and Subgroup Analysis of the BCaSS Model

To determine whether the serum BCaSS model can distinguish bladder cancer from OCa, BDs, and HPs, we calculated the discrimination index of the BCaSS model in different diseases (Figure S2 A–C). A discrimination index ≥ 0.5 was determined as BCa, and a discrimination index < 0.5 was determined as NBCa. The results showed that the BCaSS model has excellent discrimination ability for BCa and other 12 tumors, BDs, and HPs. The results of screening ACC calculation showed as radar chart (Figure S2 D–F, Table S6).

To determine the screening accuracy of the BCaSS model in different clinical subgroups of BCa, we calculated the screening index of the BCaSS model in male and female, age < 65 and age ≥ 65, Stage I and Stage II–IV subgroups (Figure S2 G–I). The BCaSS model showed excellent screening performance in different clinical subgroups of BCa. The result of screening accuracy calculation showed as radar chart (Figure S2 J–L, Table S7).

### 3.8 Evaluate the Screening Efficiency of BCaS3miR and BCaSS Using an External Set

**FIGURE 6:**
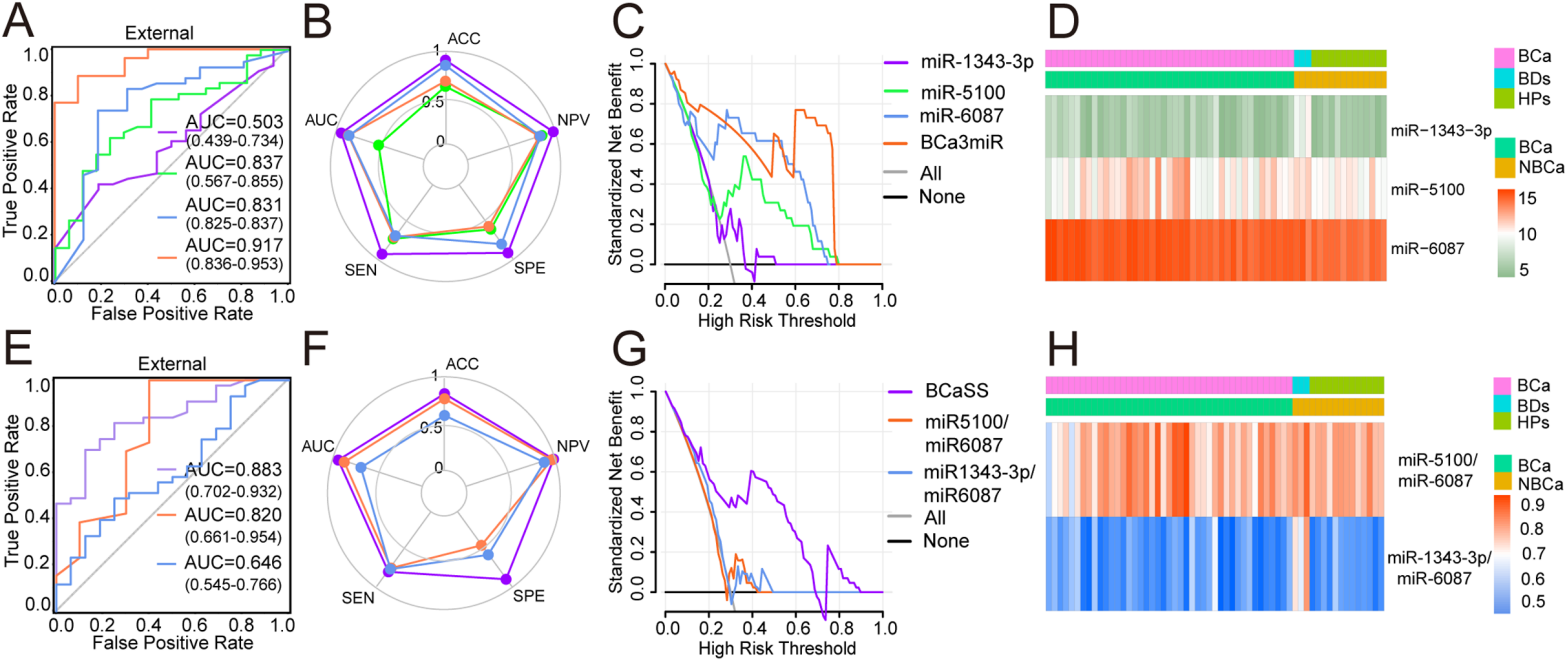
External validation of BCa screening models (A) ROC curves of BCaS3miR model (B) The radar chart summarized the ability of BCaS3miR model to recognize BCa, which were determined by ACC, AUC, SEN, SPE, and NPV (C) In a wide range of decision threshold probability, the net benefit of BCaS3miR model (D) Heatmaps of miR‐1343‐3p, miR‐5100, and miR‐6087 (E) ROC curves of BCaSS model (F) The radar chart summarized the ability of BCaSS model to recognize BC (G) In a wide range of decision threshold probability, the net benefit of BCaSS model (H) Heatmaps of miR‐1343‐3p/miR‐6087 and miR‐5100 /miR‐6087.

To further evaluate the screening performance of the model, both the BCaS3miR and BCaSS models were validated using the external dataset (GSE201359). This dataset was developed by Toray Industries, Inc., Japan. It was published in the GEO database in 2022 using the GPL21263 3D‐Gene Human miRNA V21_1.0.0 platform. The AUC value for BCaS3miR (Figure 6A) was 0.917 (95% CI: 0.836–0.953), while the AUC value for BCaSS (Figure 6E) was 0.883 (95% CI: 0.702–0.932). The radar chart provided an overview of the ACC, AUC, SEN, SPE, and NPV for BCaS3miR (Figure 6B) and BCaSS (Figure 6F). The DCA demonstrated the remarkable net benefit of both BCaS3miR (Figure 6C) and BCASS (Figure 6G) models across a wide range of decision‐making threshold probabilities. The heatmap clustering (Figure 6D) displayed the values of miR‐1343‐3p, miR‐5100, and miR‐6087 for each sample, while Figure 4H presented the values of miR‐1343‐3p/miR‐6087 and miR‐5100/miR‐6087 for each sample. These results clearly indicate that both the BCaS3miR and BCaSS models exhibit excellent predictive efficacy and stability when applied to the external dataset.

## 4 Discussion

In this study, we developed screening models for BCa by employing five machine learning algorithms (KNN, SVC, XGBT, RFC, and LR). The diagnostic performance of all models was evaluated based on the validation set, and the optimal diagnostic model (BCaS3miR) was obtained. The model was constructed using the largest sample size and the most complex mixed cohort known for serum miRNA‐based BCa diagnosis. The sample comprised 16,189 participants, including individuals diagnosed with BCa, other cancers (OCa), benign diseases (BDs), and healthy participants (HPs).

The BCaS3miR model contains only three miRNA features and had an overall sensitivity of 92.2% and a specificity of 94.4%. The newly developed model demonstrates superior performance compared with existing noninvasive BCa diagnostic methods that are currently approved by the FDA. These include BTAstat, BTAtrak, NMP22, FDP, ImmunoCyt, FISH, and urine cytology [6, 13, 14, 15]. These enhanced diagnostic capabilities make it a potentially more reliable and accurate tool for early detection and monitoring of bladder cancer, reducing the likelihood of false positives and negatives often associated with existing methods. In comparison with the traditional methods like urine cytology, which often struggles with low sensitivity for low‐grade tumors, or tests like FISH, which can produce variable results depending on the tumor's genetic makeup, this model offers a more consistent and reliable approach. The BCaS3miR model has excellent discrimination power for BCa, OCa, BDs, and HPs based on the index calculation results. Clinical subgroup analysis further supports that the model has excellent screening accuracy across different BCa clinical subgroups, affirming generalizability. Our model showed very stable and exceptional BCa screening performance in the mixed cohort, approaching to clinical practice.

Currently, there have been studies on using liquid biopsies for noninvasive detection of bladder cancer based on large sample‐sized biomarker selection. This includes detection BCa through detecting miRNA, DNA mutations, and DNA chemical modifications in patients' serum or urine [6, 15]. Recent studies on the urinary microbiome suggest a significant interplay with bladder cancer. Specifically, Porphyromonas somerae has been identified as a potential microbial biomarker in male patients over 50 [16]. Due to its stability and tumor specificity in biological fluids, miRNA expression is extensively studied as a diagnostic biomarker for cancer, especially for fluid miRNAs [7, 17, 18]. Although studies suggest that urine miRNA detection leads to good performance [13, 19], the level of miRNA in urine is easily affected by various factors such as exfoliated uroepithelial cells, urinary tract infections, and hematuria, making it challenging to use in clinical practice to diagnose BCa. A meta‐analysis revealed that using blood miRNA for BCa detection yields better accuracy than urine miRNA [20]. Reports have suggested that bladder cancer screening can be made through serum miRNA microarray or next‐generation sequencing results [21]. These studies only considered BCa patients and healthy individuals as research participants, overlooking the significance of miRNAs' specificity for BCa screening in mixed cohorts including BCa, multiple other tumors, benign disease patients, and normal individuals. This study's design does not align with the population characteristics encountered during large‐scale screening for BCa. The novelty of our study is that we built a BCa screening model using a mixed cohort of studies with the largest sample size reported to date and the most types of cancer and disease. Our BCaS3miR model can differentiate BCa from the 12 OCa, BDs, and HPs in mixed cohorts. Compared with existing models [10], our model has significantly better screening ability for BCa and accuracy in distinguishing OCa in mixed cohorts. Early‐stage bladder cancer occurs in uroepithelial cells of the urinary tract and has not invaded the muscle layer of the bladder tissue. The screening accuracy of the model in the Stage I subgroup directly reflects the early‐stage BCa screening ability of the model. Achieving an accuracy > 95% in both the Stage I and Stage II–IV subgroups is a testament to the screening performance of the BCaS3miR model. The BCaS3miR model shows potential for early‐stage BCa screening and is projected to deliver exceptional screening results.

The BCaS3miR model constructed in our study comprises three markers, including miR‐1343‐3p, miR‐5100, and miR‐6087, that can detect BCa. Previous studies have shown that serum miR‐6087 can be used as detection markers for BCa, and achieve good detection efficacy. Yuan et al. [22] examined the levels of circulating extracellular RNA in 50 healthy individuals and 142 cancer patients and revealed the association between the incidence of cancer and the downregulation of miR‐1343‐3p. However, no study has reported the involvement of miR‐5100 in BCa, and additional molecular mechanism investigations are necessary to clarify their biological functions in BCa. In our study that used a single miRNA to construct a BCa screening model based on machine learning algorithms, we found that regardless of the algorithm used, miR‐6087 showed high screening efficacy for BCa. The AUC values of all models were greater than 0.8, which was similar to the remarkable report by Usuba et al. [10] However, the current research on the correlation between miR‐6087 and the occurrence and development mechanism of BCa is still lacking, and future basic experiments need to be conducted to explore its specific mechanism. Our established BCa screening model is expected to obtain the expression levels of miRNA biomarkers in serum using real‐time quantitative PCR. By detecting the expression of serum miR‐1343‐3p, miR‐5100, and miR‐6087, can judge whether patients suffering from BCa, and can provide clinical test report in 1 day. The discovery of bacterial signatures, such as the increased abundance of Porphyromonas and Porphyromonas somerae [16], does coincide with the growing interest in noninvasive diagnostics. Combining microbiome analysis with miRNA profiles can improve the predictive power and specificity of BCa screening models. Urinary micrornas have been extensively studied as biomarkers of urinary system cancers [23]. Combining serum and urine markers may provide a promising integrated approach for early BCa detection.

In addition, the batch effects and technical differences in sequencing can affect the sequencing results of serum miRNAs, leading to changes in the weight of each miRNA in the constructed model, which in turn affects the predictive performance of the model. Ratio‐based predictive models can address these issues. For that reason and to meet the need for a good normalization factor for serum samples, we hypothesized that use of a combination (as a ratio) of an overexpressed and an underexpressed miRNA in the same RNA sample from a BCa patient could be a good within‐sample normalization method [24]. Cabrera et al. [4] successfully built a ratio model for urine miRNAs (miR‐145/miR‐182), with favorable predictive accuracy and stability. In this study, a serum miRNA score model (BCaSS) was established for the screening model of BCa. The BCaSS model is the first bladder cancer screening model that utilizes serum miRNA ratio. Screening performance analysis revealed that the BCaSS model demonstrated excellent predictive performance, with an AUC value of 0.967. In addition, when compared to the BCaS3miR bladder cancer screening model built in this study, the predictive performance of the BCaSS model is slightly inferior. But the BCaSS model has unique advantages.

There are still some limitations. While our study was founded on public data and was not clinically tested, we initiated a prospective clinical study to evaluate the screening efficacy of the BCaS3miR model in clinical practice. We are actively considering follow‐up research that will involve tracking participants over time to observe the evolution of the screening models' accuracy and generalizability in dynamic, real‐world clinical settings. Demographic variations and comorbid conditions are important factors that could affect the performance of our screening models. Further exploration through molecular biology experiments is still necessary to understand the biological mechanisms of the serum miRNAs incorporated in developing the BCaS3miR model for BCa screening. Only one external cohort was used to validate the model, the repeatability of the model to study to further improve in the future.

## 5 Conclusions

In summary, we developed a noninvasive BCa screening model using serum miRNAs based on sequencing data derived from 16,189 samples in a mixed cohort. The model demonstrates impressive screening accuracy and great potential for early detection of BCa. Moreover, a prospective multicenter clinical study across several large hospitals is planned to assess the potential clinical application of our developed models in screening for BCa.

## Author Contributions

**Cong Lai:** data curation (equal), methodology (equal), validation (equal). **Zhensheng Hu:** data curation (equal), writing – original draft (equal). **Jintao Hu:** visualization (equal). **Zhuohang Li:** funding acquisition (equal). **Lin Li:** investigation (equal). **Mimi Liu:** investigation (equal). **Zhikai Wu:** visualization (equal). **Yi Zhou:** supervision (equal). **Cheng Liu:** writing – review and editing (equal). **Kewei Xu:** visualization (equal).

## Ethics Statement

The authors have nothing to report.

## Consent

The authors have nothing to report.

## Conflicts of Interest

The authors declare no conflicts of interest.

## Supporting information


Appendix S1.

## Data Availability

The datasets used and/or analyzed during the current study can be made available from the corresponding author upon reasonable request.
