# Supplementary material for: Screening Model for Bladder Cancer Early Detection With Serum miRNAs Based on Machine Learning: A Mixed‐Cohort Study Based on 16,189 Participants
Source: Cancer Med. 2024 Oct 23;13(20):e70338. doi: 10.1002/cam4.70338 (PMC11496983; doi:10.1002/cam4.70338)
Supplement: Supplementary file 1 — Appendix S1. [file CAM4-13-e70338-s001.docx]

**
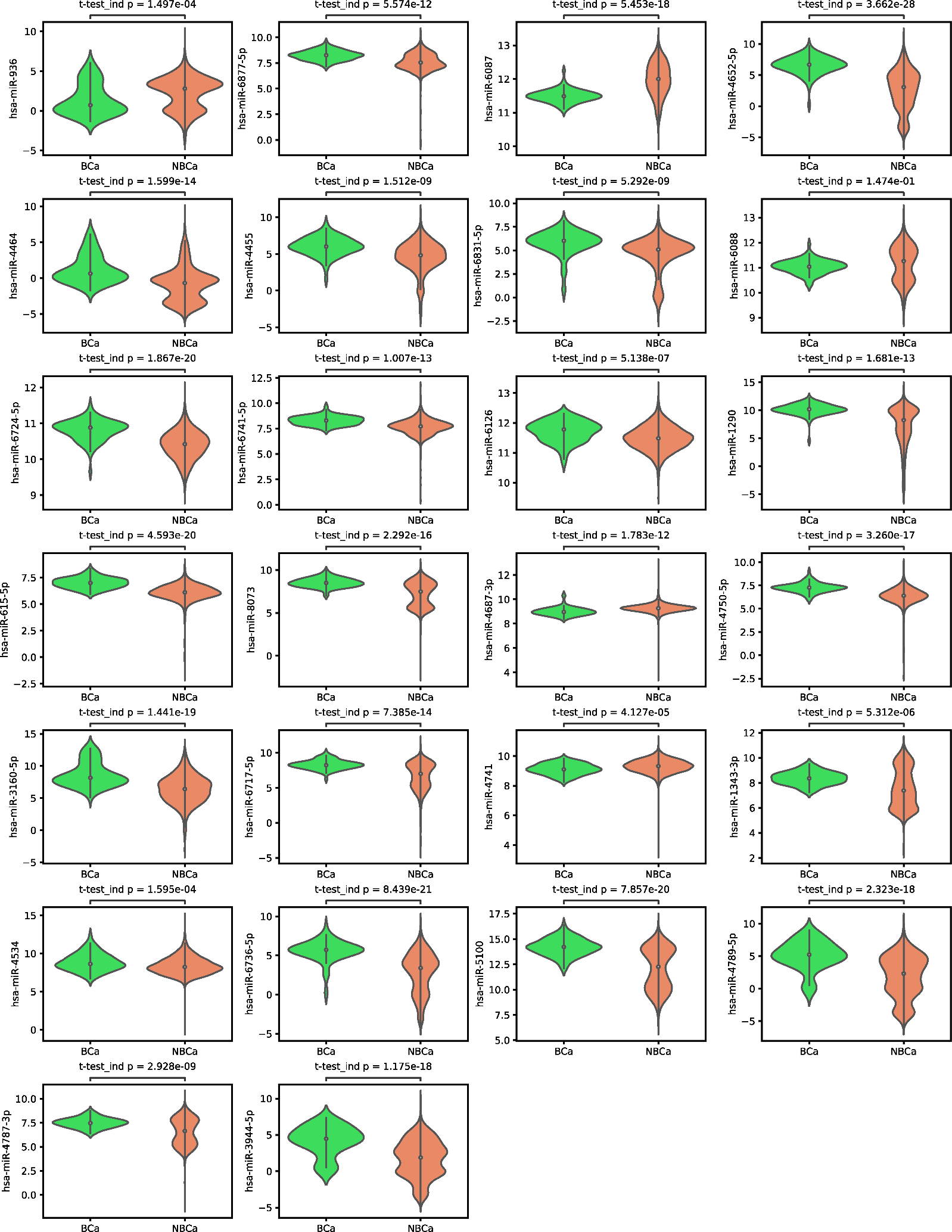
**

**FIGURE S1. The differences of 26 candidate miRNAs in BCa and NCBa**

**TABLE S1.** The five-fold cross-validation results of the BCa screening model based on five machine learning algorithms were established by using three miRNA features.

| Fold | method | ACC | AUC | SEN | SPE | NPV |
| --- | --- | --- | --- | --- | --- | --- |
| 1 | XGBT | 0.95 | 0.93 | 0.86 | 0.96 | 0.996 |
| 1 | RF | 0.93 | 0.97 | 0.84 | 0.93 | 0.996 |
| 1 | SVM | 0.96 | 0.98 | 0.88 | 0.96 | 0.99 |
| 1 | KNN | 0.97 | 0.95 | 0.92 | 0.97 | 0.99 |
| 1 | LR | 0.88 | 0.94 | 0.86 | 0.88 | 0.996 |
| 2 | XGBT | 0.94 | 0.96 | 0.95 | 0.94 | 0.99 |
| 2 | RF | 0.91 | 0.97 | 0.92 | 0.90 | 0.99 |
| 2 | SVM | 0.96 | 0.98 | 0.95 | 0.96 | 0.99 |
| 2 | KNN | 0.97 | 0.96 | 0.94 | 0.97 | 0.99 |
| 2 | LR | 0.86 | 0.95 | 0.92 | 0.86 | 0.99 |
| 3 | XGBT | 0.93 | 0.99 | 1.00 | 0.93 | 1.00 |
| 3 | RF | 0.93 | 0.99 | 1.00 | 0.93 | 1.00 |
| 3 | SVM | 0.97 | 0.99 | 1.00 | 0.97 | 1.00 |
| 3 | KNN | 0.97 | 0.99 | 0.97 | 0.97 | 0.99 |
| 3 | LR | 0.88 | 0.97 | 0.97 | 0.88 | 0.99 |
| 4 | XGBT | 0.91 | 0.97 | 0.90 | 0.91 | 0.99 |
| 4 | RF | 0.91 | 0.97 | 0.90 | 0.91 | 0.99 |
| 4 | SVM | 0.96 | 0.99 | 0.94 | 0.96 | 0.99 |
| 4 | KNN | 0.97 | 0.95 | 0.89 | 0.97 | 0.99 |
| 4 | LR | 0.88 | 0.95 | 0.92 | 0.87 | 0.99 |
| 5 | XGBT | 0.96 | 0.96 | 0.94 | 0.96 | 0.99 |
| 5 | RF | 0.93 | 0.97 | 0.94 | 0.93 | 0.99 |
| 5 | SVM | 0.96 | 0.99 | 0.94 | 0.96 | 0.99 |
| 5 | KNN | 0.97 | 0.96 | 0.89 | 0.98 | 0.99 |
| 5 | LR | 0.88 | 0.97 | 0.95 | 0.87 | 0.99 |

**TABLE S2. The AUC, SEN, SPE, ACC and NPV of the BlaS3miR model in the training set, testing set and validation set.**

|  | ACC | AUC | SEN | SPE | NPV |
| --- | --- | --- | --- | --- | --- |
| Training set | 1.00 | 1.00 | 1.00 | 1.00 | 1.00 |
| Testing set | 0.97 | 0.99 | 0.97 | 0.97 | 0.99 |
| Validation set | 0.97 | 0.96 | 0.91 | 0.97 | 0.99 |

**TABLE S3. ACC of BCaS3miR model distinguish BCa from OCa, BDs and HPs.**

|  | BCa | LCa | CCa | GCa | PCa | PC | BRCa | ECa | BTCa | OC | HCC | Sarcoma | Glioma | BDs | HPs |
| --- | --- | --- | --- | --- | --- | --- | --- | --- | --- | --- | --- | --- | --- | --- | --- |
| Training set | 1.00 | 1.00 | 1.00 | 1.00 | 1.00 | 1.00 | 1.00 | 1.00 | 1.00 | 1.00 | 1.00 | 1.00 | 1.00 | 1.00 | 1.00 |
| Testing set | 0.97 | 0.98 | 0.96 | 0.99 | 0.83 | 1.00 | 1.00 | 0.98 | 0.89 | 0.89 | 1.00 | 1.00 | 0.89 | 0.99 | 0.99 |
| Validation set | 0.91 | 0.97 | 0.98 | 0.99 | 0.81 | 1.00 | 1.00 | 0.99 | 0.86 | 0.91 | 1.00 | 1.00 | 0.85 | 0.98 | 0.99 |

**TABLE S4. ACC of BCaS3miR model diagnostics BCa in the different subgroups.**

|  | Male | Female | <65 | ≥65 | Stage I | Stage II-IV |
| --- | --- | --- | --- | --- | --- | --- |
| Training set | 1 | 1 | 1 | 1 | 1 | 1 |
| Testing set | 1 | 0.95 | 1 | 0.95 | 1 | 0.95 |
| Validation set | 0.85 | 0.93 | 0.84 | 0.94 | 1 | 0.92 |


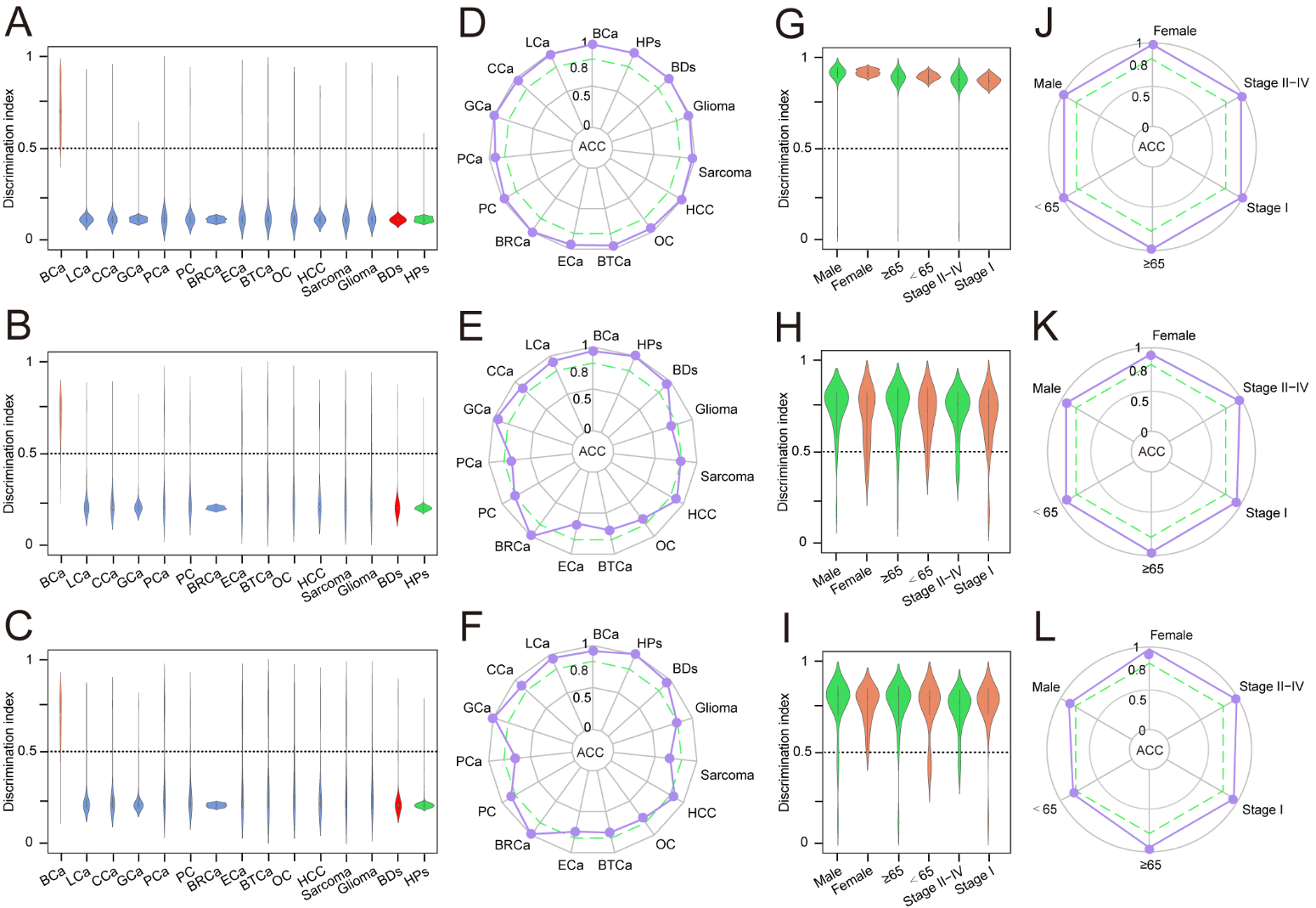


**FIGURE S2. Screening performance and subgroup analysis of the BCaSS model.** Discrimination index was calculated and plotted in a dot plot among BCa, 12 OCa, BDs and HPs for the discriminants in training set (A), testing set (B) and validation set (C). Screening index ≥0.5 indicated BCa and Screening index <0.5 indicated NBCa. The radar chart summarized the ACC of each cancer type, purple polyline represented the ACC value of BCaSS model in distinguishing each cancer in training set (D), testing set (E) and validation set (F). Screening index was calculated and plotted in a dot plot among BCa in different clinical subgroups for the discriminants in training set (G), testing set (H) and validation set (I). The radar chart summarized the ACC of each subgroup, purple polyline represented the ACC value of BCaSS model in distinguishing each subgroup in training set (J), testing set (K) and validation set (L).

**TABLE S5. The AUC, SEN, SPE, ACC and NPV of the BlaSS model in the training set, testing set and validation set.**

|  | ACC | AUC | SEN | SPE | NPV |
| --- | --- | --- | --- | --- | --- |
| Training set | 1.00 | 0.99 | 1.00 | 1.00 | 1.00 |
| Testing set | 0.91 | 0.97 | 0.95 | 0.91 | 0.99 |
| Validation set | 0.92 | 0.97 | 0.93 | 0.92 | 0.99 |

**TABLE S6. ACC of BCaSS model distinguish BCa from OCa, BDs and HPs.**

|  | BCa | LCa | CCa | GCa | PCa | PC | BRCa | ECa | BTCa | OC | HCC | Sarcoma | Glioma | BDs | HPs |
| --- | --- | --- | --- | --- | --- | --- | --- | --- | --- | --- | --- | --- | --- | --- | --- |
| Training set | 1.00 | 0.99 | 0.97 | 1.00 | 0.93 | 0.98 | 1.00 | 0.95 | 0.96 | 0.95 | 0.99 | 0.96 | 0.98 | 1.00 | 1.00 |
| Testing set | 0.95 | 0.93 | 0.90 | 0.98 | 0.72 | 0.82 | 1.00 | 0.63 | 0.71 | 0.75 | 0.91 | 0.81 | 0.74 | 0.96 | 1.00 |
| Validation set | 0.93 | 0.94 | 0.88 | 0.99 | 0.67 | 0.87 | 1.00 | 0.75 | 0.76 | 0.78 | 0.89 | 0.68 | 0.81 | 0.95 | 1.00 |

**TABLE S7. ACC of BCaSS model diagnostics BCa in the different subgroups.**

|  | Male | Female | <65 | ≥65 | Stage I | Stage II-IV |
| --- | --- | --- | --- | --- | --- | --- |
| Training set | 1.00 | 1.00 | 1.00 | 1.00 | 1.00 | 1.00 |
| Testing set | 0.95 | 0.95 | 0.95 | 0.97 | 0.96 | 1.00 |
| Validation set | 1.00 | 0.90 | 0.84 | 0.96 | 0.96 | 1.00 |
